# Modelling Population Dynamics in Realistic Landscapes with Linear Elements: A Mechanistic-Statistical Reaction-Diffusion Approach

**DOI:** 10.1371/journal.pone.0151217

**Published:** 2016-03-17

**Authors:** Lionel Roques, Olivier Bonnefon

**Affiliations:** INRA, UR 546 BioSP, 84914 Avignon, France; Shanxi University, CHINA

## Abstract

We propose and develop a general approach based on reaction-diffusion equations for modelling a species dynamics in a realistic two-dimensional (2D) landscape crossed by linear one-dimensional (1D) corridors, such as roads, hedgerows or rivers. Our approach is based on a hybrid “2D/1D model”, i.e, a system of 2D and 1D reaction-diffusion equations with homogeneous coefficients, in which each equation describes the population dynamics in a given 2D or 1D element of the landscape. Using the example of the range expansion of the tiger mosquito *Aedes albopictus* in France and its main highways as 1D corridors, we show that the model can be fitted to realistic observation data. We develop a mechanistic-statistical approach, based on the coupling between a model of population dynamics and a probabilistic model of the observation process. This allows us to bridge the gap between the data (3 levels of infestation, at the scale of a French department) and the output of the model (population densities at each point of the landscape), and to estimate the model parameter values using a maximum-likelihood approach. Using classical model comparison criteria, we obtain a better fit and a better predictive power with the 2D/1D model than with a standard homogeneous reaction-diffusion model. This shows the potential importance of taking into account the effect of the corridors (highways in the present case) on species dynamics. With regard to the particular case of *A. albopictus*, the conclusion that highways played an important role in species range expansion in mainland France is consistent with recent findings from the literature.

## Introduction

A common feature of many human-modified landscapes is that they are made up mainly of two-dimensional (2D) patches, such as fields or forest stands, and narrow linear (or near-linear) one-dimensional (1D) elements, such as roads or hedgerows. These narrow elements can have a strong influence on the dynamics of species that live in such environments, as illustrated by the well-documented “corridor effect”: it has been shown that such linear elements tend to increase movement between habitat patches for a diverse set of organisms and across a wide range of ecosystems [1, 2]. By promoting individual movements, corridors can contribute to the spread of pest species, as in the recent range expansion of the pine processionary moth in northern France [3, 4] or the spread of the invasive species *Aedes albopictus* in metropolitan France [5], which are both suspected to be facilitated by the presence of roads. Linear elements can also impede species movement, as they can constitute barriers to dispersal [6, 7], leading to potentially important demographic and genetic consequences (see [8] and references therein).

In this paper, we consider a broader notion of corridor than the classical one in [9], who defined corridors as linear habitats embedded in a dissimilar matrix that connect two or more larger blocks of habitat. In our case, *a corridor* designates any type of narrow near-linear element of the landscape that may have a significant effect on a species dynamics. Corridors may consist of nonhabitat regions (e.g., roads) or conversely may be more favourable for the species than the rest of the landscape (e.g., riparian corridors); they may also play the role of barriers. The rest of the landscape is referred to as *the matrix*.

The local effects of landscape on individual mobility and fitness can be captured by 2D spatially explicit models, at the scale of individual movement such as in random walk models, stochastic differential equations, and models of branching random walks [10, 11], or at the scale of population density in reaction-diffusion models [12–15]. To take into account such local effects of the landscape, position-dependent mobility and reproduction parameters are usually introduced in the models. However, such approaches may be unadapted to model the effect of narrow elements such as corridors at the scale of the landscape, e.g., at the regional scale or the country scale.

In this paper, we used a reaction-diffusion framework to model species dynamics in a landscape defined as a collection of homogeneous 2D patches constituting the matrix and separated by 1D corridors. The reaction-diffusion model is a system of equations with homogeneous coefficients, with 2D equations for the population dynamics in the patches and 1D equations for the dynamics in the corridors. This approach is adapted from [16], where a mixed 2D/1D mathematical model was proposed for describing the spread of a population in a 2D space including a unique infinite corridor (a straight line) with faster diffusion. This idealised approach showed that the presence of a corridor increased the speed of expansion of a population in the direction of the corridor if and only if the diffusion coefficient was at least twice as large in the corridor as in the matrix, demonstrating that the presence of a corridor can have a significant effect on the behaviour of the model.

Our work aims (i) to extend the model proposed by Berestycki et al. [16] to more realistic landscapes made of several 2D patches separated by 1D corridors and to include possible barrier effects; (ii) to propose a mechanistic-statistical framework [14, 17–22] combining a 2D/1D model with a probabilistic model able to deal with realistic discrete observation data; (iii) to establish an efficient algorithm for providing numerical descriptions of landscapes, and solving the corresponding systems of 2D/1D reaction-diffusion equations; (iv) to apply this framework to the range expansion of the tiger mosquito *A. albopictus* in France.

## Description of the 2D/1D model

We consider a landscape made of a 2D matrix crossed by 1D corridors, e.g., hedgerows or roads. The main idea behind our approach is to describe the dynamics of a species in the matrix and in the corridors by two different models: the dynamics in the matrix are described by a set of 2D reaction-diffusion equations, and the dynamics in the corridors by another set of 1D reaction-diffusion equations. The exchanges between the matrix and the corridors are described by coupling terms between the two sets of equations.

One of the simplest cases is a matrix crossed by a single corridor. This is the situation which was considered in the original model of Berestycki et al. [16]. More precisely, they considered an infinite matrix crossed by a straight corridor and they assumed a perfect symmetry between the population density on each side of the corridor. Their model then reduced to two equations, one for the population density *v*(*t*, *x*, *y*) at time *t* and position (*x*, *y*) in the matrix, and the other for the population density *u*(*t*, *x*) at time *t* and position *x* in the corridor:

$$\left\{\begin{array}{cc} \partial_{t}v=d\;\Delta v+f\left(v\right), & t>0,\;(x,y)\in R\times (0,+\infty ),\\ \partial_{t}u=D\;\partial_{xx}u+\rho_{21}v\left(t,x,0\right)-\rho_{12}\;u, & t>0,\;x\in R, \end{array}\right.$$ (1)

with the boundary condition:

$$-d\partial_{y}v\left(t,x,0\right)=\rho_{12}\;u\left(t,x\right)-\rho_{21}v\left(t,x,0\right),t>0,\;x\in R.$$

Here, Δ = ∂_*xx*_ + ∂_*yy*_ is the 2D Laplace diffusion operator and ∂_*xx*_ is the 1D Laplace diffusion operator. These operators describe uncorrelated random walk movements of the individuals (for general results on reaction-diffusion models, see [12, 15, 23–25]). The mobility of the individuals can be influenced by their position: this is described by the coefficients *d* and *D*, which measure the mobility of the individuals in the matrix and in the corridor, respectively. The coefficient *ρ*_12_ is the exchange rate from the corridor to the 2D matrix, and the coefficient *ρ*_21_ is the exchange rate from the 2D matrix to the corridor. The function *f* describes population growth in the matrix, whereas no growth was assumed in the corridor.

### Domain

**Fig 1:**
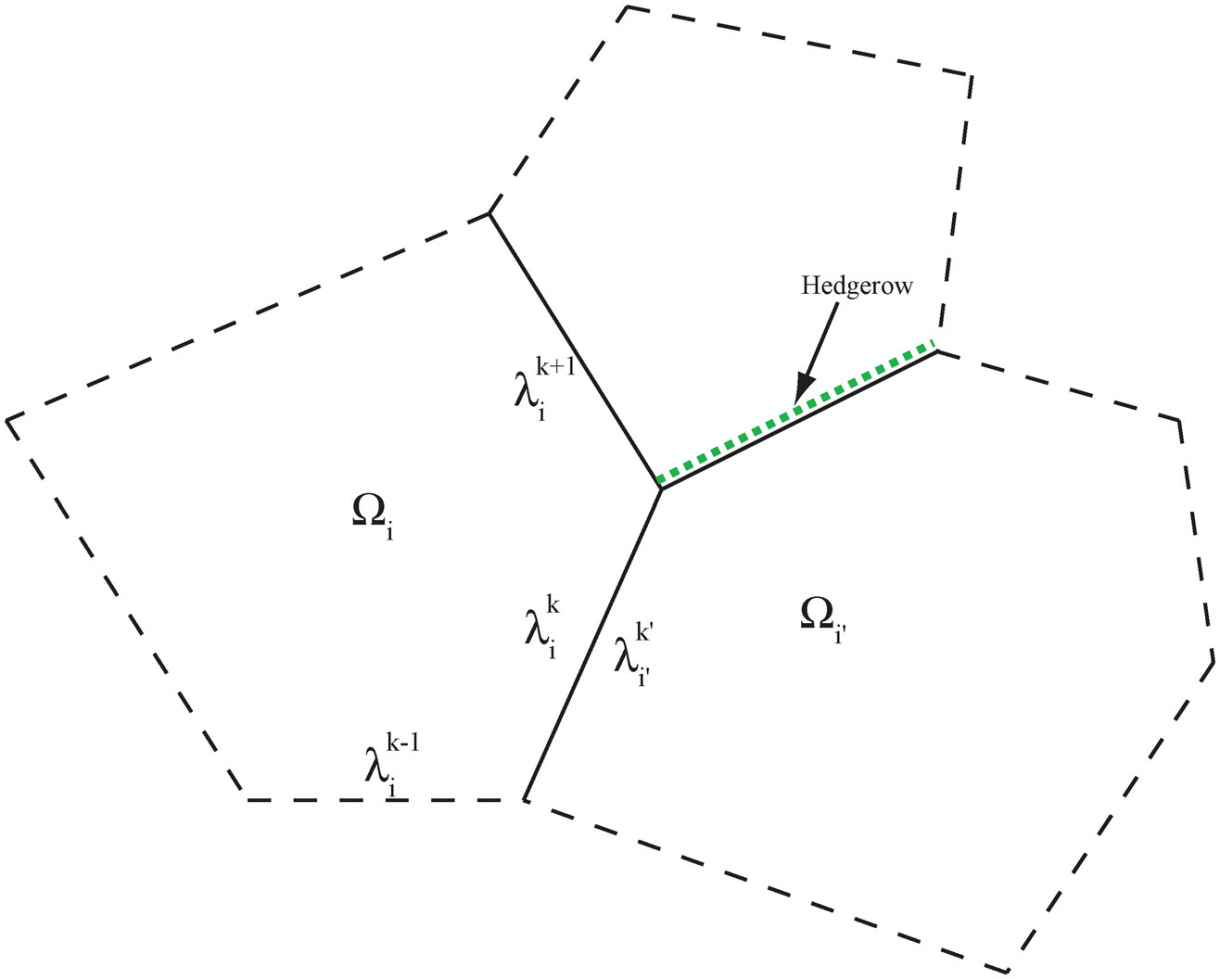
Example of a landscape made of three 2D patches. Solid lines correspond to interior edges (= corridors), and dashed lines to exterior edges with no 1D dynamics. Interior edges may be asymmetrical, for instance with a wall on the one side and a hedgerow on the other. The indices *k* are taken anti-clockwise.

We consider a 2D matrix defined by a set Ω ⊂ ℝ^2^, made of a finite mosaic of *I* polygonal disjoint 2D patches *Ω*_*i*_ separated by corridors (Fig 1). The boundary of the patches are denoted by ∂*Ω*_*i*_, each boundary consisting of a finite number of 1D edges $\lambda_{i}^{k}$. We assume that the edges fall into two categories: the interior edges (= the corridors), and the exterior edges which belong to the boundary ∂*Ω* of *Ω* and where no particular 1D dynamics is modelled. Another category, which is not described here for the sake of simplicity although used in our numerical computations, corresponds to the corridors that belong to the boundary ∂*Ω* of *Ω*.

### Population densities

In each 2D patch *Ω*_*i*_ of the matrix, the population density is denoted by *v*_*i*_. The population density in any corridor $\lambda_{i}^{k}$ is denoted by $u_{i}^{k}$.

### Dynamics in the matrix

In each patch *Ω*_*i*_ of the matrix, the population density is modelled by a reaction-diffusion equation:

$$\partial_{t}v_{i}=d\;\Delta v_{i}+f\left(v_{i}\right),$$ (2)

with a diffusion parameter *d* which measures the mobility of the individuals in the matrix, and a growth function *f* which describes birth and death events in the patch *Ω*_*i*_. The function *f* may depend on the position to account for spatial heterogeneities in the birth and death rates; see e.g. [24] for examples of typical growth functions. The effect of the exchanges between each patch *Ω*_*i*_ and the surrounding corridors are described by the fluxes terms:

$$d\;\nabla v_{i}\cdot n=\rho_{12}u_{i}^{k}\left(t,x,y\right)-\rho_{21}v_{i}\left(t,x,y\right),$$ (3)

where $\rho_{12}u_{i}^{k}\left(t,x,y\right)$ describes the flux of individuals leaving the corridor $\lambda_{i}^{k}$ and entering the patch *Ω*_*i*_ at time *t* and at the position (*x*, *y*) and $\rho_{21}v_{i}\left(t,x,y\right)$ describes the flux of individuals leaving the patch *Ω*_*i*_ and entering the corridor $\lambda_{i}^{k}$. The coefficients *ρ*_12_ and *ρ*_21_ may also vary from one corridor to another to account for possible differences in the nature of the corridors and subsequent effects on the transfer rates. Here, **n** = **n**(*x*, *y*) denotes the outward unit normal to the boundary ∂*Ω*_*i*_. On the exterior boundary edges $\lambda_{i}^{k}\in \partial \Omega ,$ standard reflecting boundary conditions are assumed [13, 15]:

$$\nabla v_{i}\cdot n=0.$$ (4)

The boundary conditions Eq (4) mean that either the individuals crossing the boundaries are reflected inside the domain (e.g., on coasts) or that the inward and outward fluxes are equal (other mainland boundaries). Absorbing conditions (*v*_*i*_ = 0), could be considered as well. They mean that all of the individuals which cross the boundaries instantaneously disappear.

### Dynamics in the corridors

**Fig 2:**
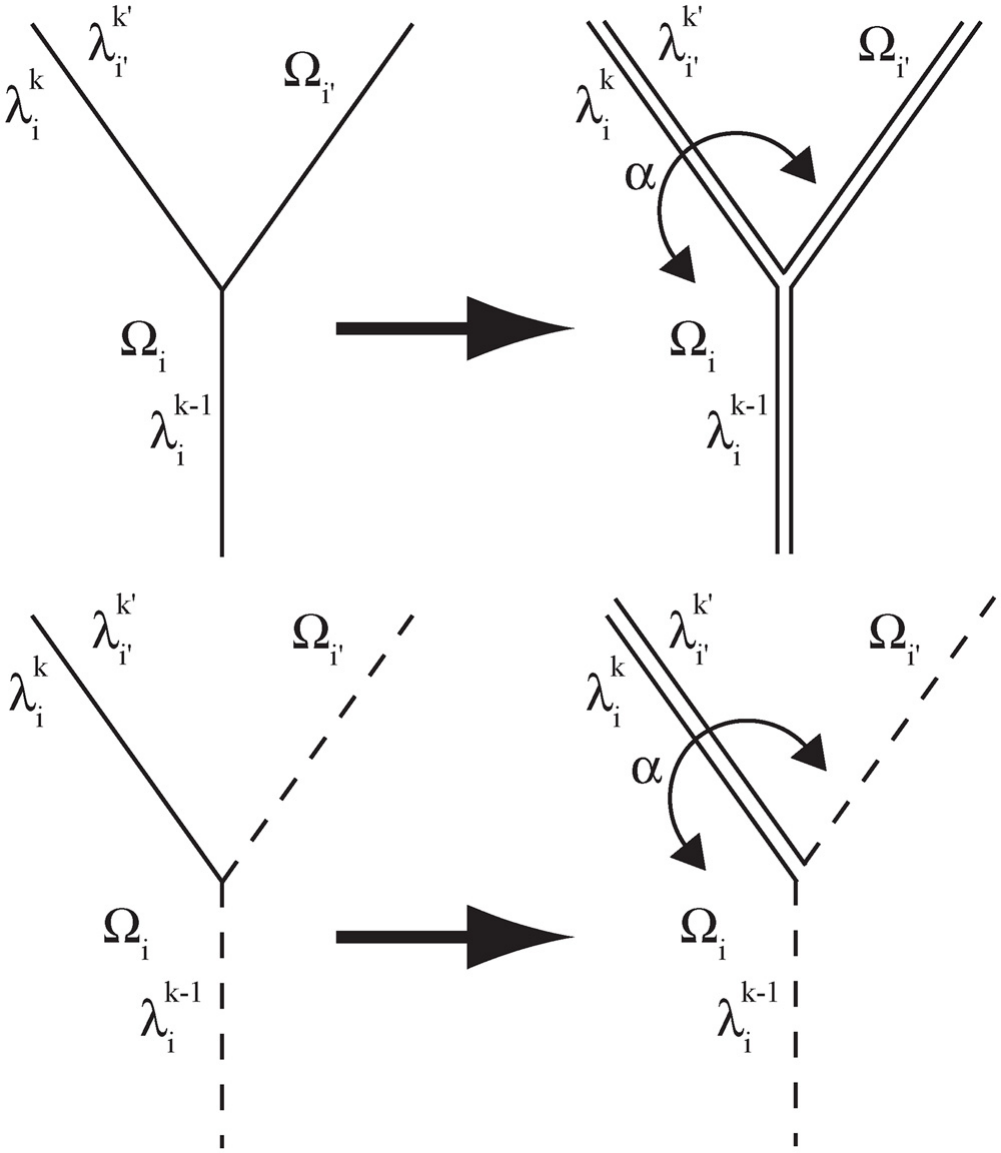
The *Y*-shaped edge junction issue. Left: the description of the population fluxes between $\lambda_{i}^{k-1}$ and $\lambda_{i}^{k}$ is not trivial due to the *Y*-shaped edge junction. Right: each interior edge (= corridor) has been duplicated (at the same position), leading to a “*V*-shaped” junction between $\lambda_{i}^{k-1}$ and $\lambda_{i}^{k}$. Solid lines correspond to interior edges. The exchange rate between the populations from both sides of the edge, $\lambda_{i}^{k}=\lambda_{i^{\prime }}^{k^{\prime }}$, are described by the parameter *α*.

One of the main difficulties in extending the approach proposed by [16] to more general domains made of several 2D patches is how to describe population fluxes at *Y*-shaped corridor junctions, or more generally at vertices connecting more than two corridors. To overcome this difficulty, we first note that each corridor $\lambda_{i}^{k}$ belongs to the common boundary of *Ω*_*i*_ and of another set, which is denoted by *Ω*_*i*′_, i.e., $\lambda_{i}^{k}=\lambda_{i^{\prime }}^{k^{\prime }}$ from a geometrical viewpoint. In some situations, for instance if the corridor is asymmetrical (e.g., a wall on the one side and a hedgerow on the other side, see Fig 1), the 1D dynamics can be different on each side of the corridor. Hence, the population densities in the corridor, seen from the *Ω*_*i*_ side and the *Ω*_*i*′_ side respectively, are denoted by $u_{i}^{k}$ and $u_{i^{\prime }}^{k^{\prime }}$, and we assumed that $u_{i}^{k}\neq u_{i^{\prime }}^{k^{\prime }}$ in general. The exchanges between the two sides of the corridor are taken into account through a permeability parameter *α* > 0 (which may depend on the location). The introduction of this extra parameter, which was not present in the theoretical model of [16], not only allows to describe barrier effects, but as shown below, also leads to a simple description of the boundary conditions satisfied by the 1D population density at the vertices, because these conditions only have to describe the regularity properties between exactly two corridors, thus solving the *Y*-shaped edge junction issue; see Fig 2.

To state a simple equation for the 1D dynamics in the corridors, we define an isometric transformation *z* ↦ (*x*(*z*), *y*(*z*)) which maps any corridor *λ* into an interval (0, *L*(*λ*)), where *L*(*λ*) is the length of the corridor (this approach can be extended to more general geometries by replacing the edges $\lambda_{i}^{k}$ by arcs which are not necessarily straight-line segments, see S1 Text for more details). The population density in the new coordinate *z* ∈ (0, *L*(*λ*)) is defined by $\tilde{u}\left(t,z\right)=u\left(t,x,y\right)$. Then, in each corridor $\lambda_{i}^{k}=\lambda_{i^{\prime }}^{k^{\prime }}$ separating two patches *Ω*_*i*_ and *Ω*_*i*′_, the equation satisfied by the population density is:

$$\begin{array}{cc} \partial_{t}{\tilde{u}}_{i}^{k}= & D\;\partial_{zz}{\tilde{u}}_{i}^{k}+\rho_{21}\;v_{i}\left(t,x\left(z\right),y\left(z\right)\right)-\rho_{12}{\tilde{u}}_{i}^{k}\left(t,z\right)\\ & -\alpha {\tilde{u}}_{i}^{k}\left(t,z\right)+\alpha {\tilde{u}}_{i^{\prime }}^{k^{\prime }}\left(t,z\right)+g\left({\tilde{u}}_{i}^{k}\right),\;t>0,\;z\in \left(0,L\left(\lambda_{i}^{k}\right)\right), \end{array}$$ (5)

where *g* is the growth function in the corridor $\lambda_{i}^{k}$; for some species where the corridor acts as a refugium, one may for instance assume that *g* > *f*, meaning that the growth rate is higher in the corridors than in the matrix. The function *g* may also vary from one corridor to another to account for spatial heterogeneities.

As mentioned earlier and shown in Fig 2, each vertex corresponds to a junction between exactly two edges. At these junctions, it is necessary to impose certain boundary conditions. In particular, standard boundary conditions describe the regularity of the population density at the vertices and the conservation of mass. An introduction point with intensity *u*_*s*_ can also be modelled at any vertex by imposing a constant population density at this point during some time interval. The various cases are summarised below; consider a junction $\lambda_{i}^{k-1}-\lambda_{i}^{k}$:

- case 1: connection between two corridors. Standard boundary conditions (regularity and conservation of mass):
  $${\tilde{u}}_{i}^{k}\left(t,0\right)={\tilde{u}}_{i}^{k-1}\left(t,L\left(\lambda_{i}^{k-1}\right)\right)\;\text{and}\;\partial_{z}{\tilde{u}}_{i}^{k}\left(t,0\right)=\partial_{z}{\tilde{u}}_{i}^{k-1}\left(t,L\left(\lambda_{i}^{k-1}\right)\right);$$ (6)
  or introduction point with intensity *u_s_*:
  $${\tilde{u}}_{i}^{k}\left(t,0\right)=u_{\text{s}}\;\text{and}\;{\tilde{u}}_{i}^{k-1}\left(t,L\left(\lambda_{i}^{k-1}\right)\right)=u_{\text{s}};$$ (7)
- case 2: connection between a corridor $\lambda_{i}^{k}$ and an exterior edge $\lambda_{i}^{k-1}\in \partial \Omega$. Standard boundary conditions (reflection):
  $$\partial_{z}{\tilde{u}}_{i}^{k}\left(t,0\right)=0;$$ (8)
  or introduction point with intensity *u_s_*:
  $${\tilde{u}}_{i}^{k}\left(t,0\right)=u_{\text{s}}.$$ (9)

### Conservation of the total population

We ascertain that the proposed approach is well-posed by verifying that the total population remains constant in the absence of growth, i.e., if the growth functions satisfy *f* = 0 and *g* = 0, and in the absence of an introduction point (we recall that reflecting conditions are assumed on the exterior boundaries). In that respect, we divide the total population into two components

$$P\left(t\right)=P_{2D}\left(t\right)+P_{1D}\left(t\right),$$

where *P*_2*D*_ is the total population in the 2D patches and *P*_1*D*_ is the total population on the 1D edges. Computing $P^{\prime }\left(t\right)=P_{2D}^{\prime }\left(t\right)+P_{1D}^{\prime }\left(t\right)$ we show that *P*′(*t*) = 0 (see S2 Text) which implies that the total population remains constant.

## Application to a real dataset: the spread of Aedes albopictus in metropolitan France

An important application of our approach is in the case of a 2D landscape crossed by roads, which are modelled as 1D corridors, with a typically higher diffusion parameter on the roads than in the rest of the landscape. This approach is illustrated by fitting the 2D/1D model to the range expansion of *A. albopictus*. At a global scale, it is known that *A. albopictus* propagation has been anthropogenically facilitated [26, 27]. At a more local scale, its rapid spread in southern France was most likely facilitated by car and truck transportation, because several isolated colonies were reported along roads in the early stage of the invasion of the Alpes-Maritimes department [28, 29] (departments are administrative divisions of the French territory). Recent studies [5] based on a statistical model have also concluded that human activities are an important driver in the spread of *A. albopictus* in metropolitan France, which makes it a good candidate to illustrate the proposed approach.

### Data

A major issue associated with *A. albopictus*, in addition to its being a significant biting nuisance, is that it is involved in the transmission of several viruses, including dengue, chikungunya [30] and probably Zika virus [31]. Since 2006, to prevent and limit the circulation of these viruses, the French Ministry of Health together with the French Regional Health Agencies have set up monitoring of the whole territory, with increased surveillance of regions where the mosquito was present or was likely to become established. For this purpose, trap nests were placed in the largest cities and along main highways. Data from 1999 to 2005 are also available, although the monitoring network did not cover the whole territory at that time.

**Fig 3:**
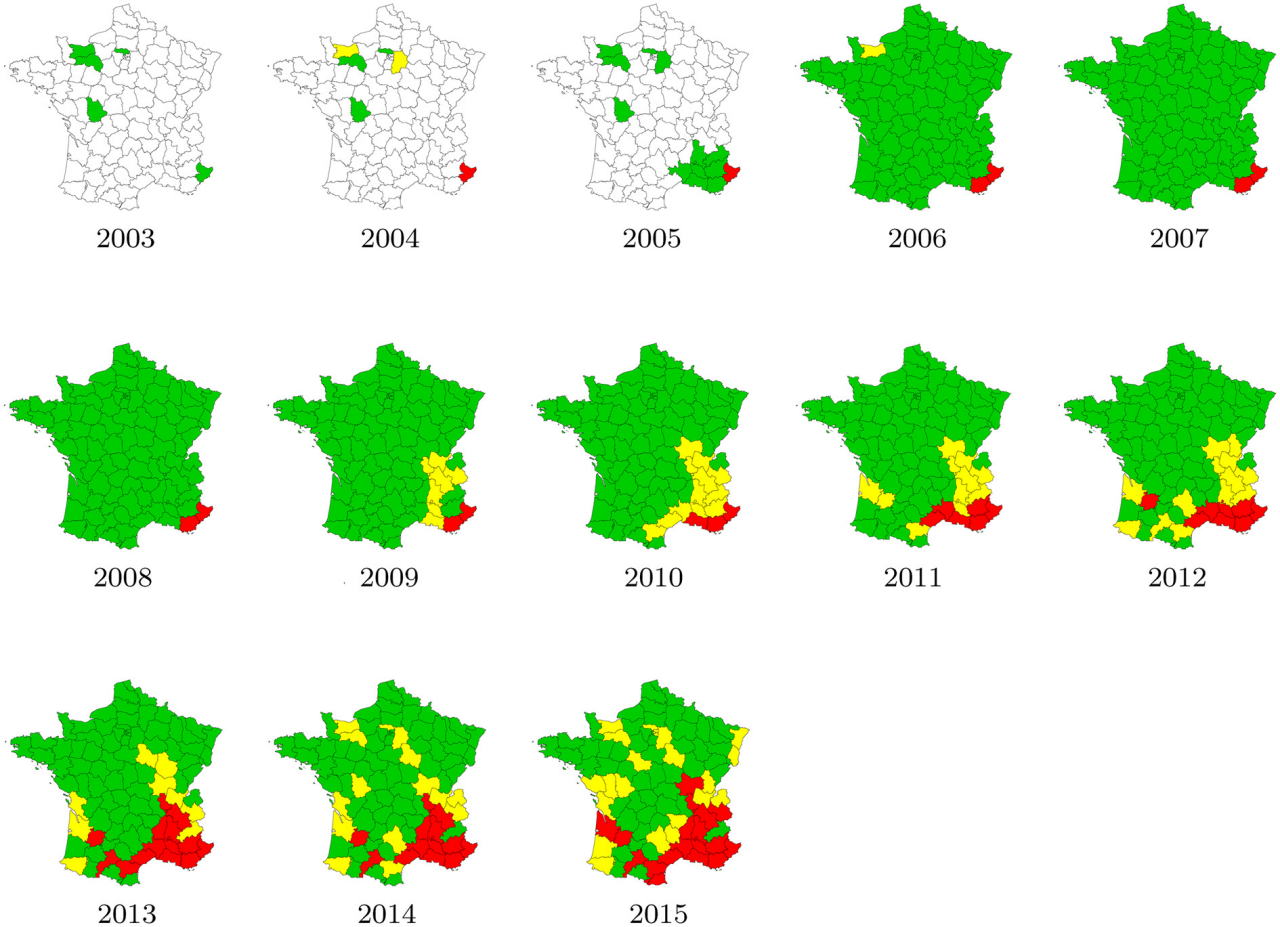
Distribution of *A. albopictus* in mainland France between 2003 and 2015. The mosquito was considered as absent in the green departments, introduced (few individuals detected) in yellow departments, and established in red departments. Before 2006, some departments were not monitored (white departments). Source: French Interdepartmental Agreement for Mosquito Control (2003–2011 data), and European Centre for Disease Prevention and Control (2012–2015 data).

The 2003–2012 data depicted in Fig 3 were available from the French Interdepartmental Agreement for Mosquito Control (EID), and the 2012–2015 data were available from the European Centre for Disease Prevention and Control (ECDC). At the scale of a French department, three levels of infestation have been defined:

- absent (green departments): field surveys were conducted and no introduction or no established population of the species have been reported;
- introduced (yellow departments): the species has been observed (but without confirmed establishment) in the administrative unit;
- established (red departments): evidence of reproduction and overwintering of the species has been observed in at least one municipality within the administrative unit.

Thus, the available data correspond, for each year *t* in 2003–2015 and each monitored department during the year *t*, to a value in {0, 1, 2}: 0 for absent; 1 for introduced; and 2 for established. This makes a total of 964 observations: 5 in 2003, 6 in 2004, 13 in 2005 and 94 each year during the period 2006–2015.

The first established population was detected in 2004 in the Alpes-Maritimes department (the south-easternmost French department), probably as a result of expansion of the Italian insect population [32]. Introductions may have also occurred from northern Spain, where it has been reported since 2004 [33]; the 2009 map suggests that such introductions may have occurred only after 2009. For the sake of simplicity, we assume a single introduction location in our model, at the French-Italian border in the Alpes-Maritimes department.

### 2D/1D model on the road map of France

**Fig 4:**
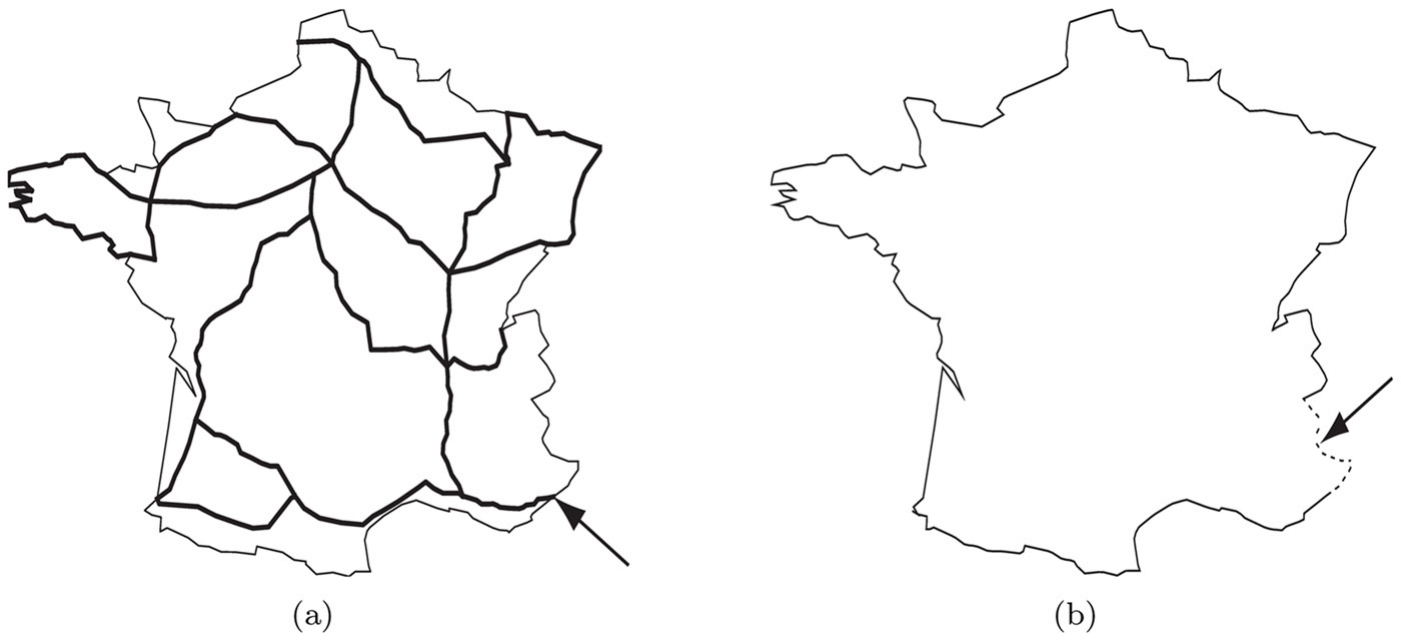
Landscapes. Left: the landscape which was used in the 2D/1D approach; the domain Ω was made up of 15 patches Ω_*i*_ separated by main highways (bold lines); the arrow corresponds to the introduction point *x_s_*. Right: the domain Ω, which was used in the classical 2D approach. The dashed region indicated by the arrow corresponds to the introduction region Γ_s_.

The 2D landscape is defined as the set *Ω* whose boundaries are those of mainland France. This landscape is made up of 15 patches *Ω*_*i*_ corresponding to the regions surrounded by highways or national boundaries (Fig 4, left). We consider only the “main highways”, i.e., the highways with an average daily traffic larger than 15000 vehicles (source: French Centre For Studies and Expertise on Risks, Environment, Mobility, and Urban and Country planning, CEREMA, 2011).

Neglecting other spatial and temporal heterogeneities, we assume a simple logistic and spatially homogeneous growth term in Eq (2), (see, e.g., [24, 25] for examples of growth functions):

$$f\left(v_{i}\right)=r\;v_{i}\left(1-\frac{v_{i}}{K}\right),$$ (10)

where *r* ≥ 0 is the intrinsic growth rate (birth rate − death rate in the absence of competition) and *K* > 0 is the carrying capacity of the environment. Assuming that the population density is expressed in units of carrying capacity, we set *K* = 1. In addition, we assume that there is no reproduction on the roads, i.e., *g* = 0 in Eq (5).

The initial condition describes the situation in 2003, where the mosquito was considered as being absent: $u_{i}^{k}\left(2003,\cdot \right)=0$ along the highways and *v*_*i*_(2003, ⋅) = 0 in all patches. An introduction point is assumed on the highway crossing the Alpes-Maritimes department (the south-easternmost French department) at its intersection with the Italian border (Fig 4, left). This situation is modelled by assuming that *u*(*t*, *x_s_*) = *u_s_* > 0 at the vertex *x_s_* corresponding to the introduction point, for all *t* ∈ [2003, 2004], see the boundary condition Eq (9); for *t* > 2004, this boundary condition is replaced by a reflecting condition Eq (8).

It is assumed that the permeability parameter *α* is known and that *α* > >1, so that the highways do not act as barriers (*α* = 1000 in our computations): the 1D densities on each side of any highway $\lambda_{i}^{k}$ are almost identical: $u_{i}^{k}≃u_{i^{\prime }}^{k^{\prime }}$ at any time and position. Finally, the unknown parameters of the 2D/1D model are:

$$\Theta =\{r,d,D,\rho_{12},\rho_{21},u_{\text{s}}\}.$$

### Classical 2D reaction-diffusion model

A classical reaction-diffusion model which does not take the road network into account is used as a benchmark (null model):

$$\left\{\begin{array}{c} \partial_{t}v=d\;\Delta v+r\;v\left(1-v\right),\;x\in \Omega ,\\ \nabla v\cdot n=0,\;x\in \partial \Omega \setminus \Gamma_{\text{s}},\\ \left\{\begin{array}{c} v=v_{\text{s}},\;\text{if}\;t\in \left[2003,2004\right]\\ \nabla v\cdot n=0\;\text{if}\;t>2004 \end{array}\right.\;x\in \Gamma_{\text{s}},\; \end{array}\right.$$ (11)

where Γ_s_ is the portion of the boundary corresponding to the French-Italian border in the Alpes-Maritimes department, corresponding to the introduction region; see Fig 4, right. Using this approach, the effect of roads is neglected. The initial condition corresponds to the absence of the mosquito: *v*(2003, *x*) = 0 for all *x* ∈ *Ω*. In this case, the only unknown parameters are

$$\Theta =\{r,d,v_{\text{s}}\}.$$

### Parameter estimation: a mechanistic-statistical approach

To bridge the gap between the data (3 levels of infestation, at the scale of a French department) and the output of the model (population densities at each point of the landscape), we use a mechanistic-statistical approach (for other examples of mechanistic-statistical models, see [14, 18–22]), based on a coupling between the model of population dynamics and a probabilistic model of the observation process. This enables us to compute a likelihood function for the parameters of the 2D/1D and the 2D model and to estimate the parameter values.

#### A probabilistic model of the observation process

We define the variable $O_{j}^{t}$ as the result of the observation in the *j*^th^ department *ω*_*j*_ ⊂ *Ω*, for *j* = 1, …, 94, at time *t*, and taking on the following values: 0 if no mosquito had been detected; 1 if a few mosquitoes had been detected; and 2 if established populations of mosquitoes had been detected (see Fig 3). We assume that the detection variables $O_{j}^{t}$ are independently drawn from a binomial distribution, conditional on the average density $V_{j}^{t}$ in the department *ω*_*j*_ at time *t*:

$$O_{j}^{t}∼B\left(2,p\left(V_{j}^{t}\right)\right),$$ (12)

where

$$V_{j}^{t}=\frac{1}{|\omega_{j}|}\int_{\omega_{j}}v\left(t,x\right)\;dx$$

is the average mosquito population density in the department *ω*_*j*_, |*ω*_*j*_| is the area of the department *ω*_*j*_, and *v*(*t*, *x*) is the mosquito population density in *Ω*, given by the 2D/1D or the 2D model. The function *p* describes the relationship between the average population density and the probability of mosquito detection. For the sake of simplicity, and because the carrying capacity is fixed to *K* = 1, a natural choice for *p* is

$$p\left(V_{j}^{t}\right)=p_{j}^{t}=\min\left(V_{j}^{t},1\right).$$ (13)

Under the assumptions of this probabilistic model, the most probable outcome is (i) the failure to detect the insect in the case of a low population density: if $p_{j}^{t}<<1$, then $O_{j}^{t}=0$ with probability ${\left(1-p_{j}^{t}\right)}^{2}≃1$; (ii) to consider the insect as being “introduced” in the case of an intermediate population density: if $p_{j}^{t}≃0.5$, then $O_{j}^{t}=1$ with probability $2p_{j}^{t}\left(1-p_{j}^{t}\right)≃0.5$; and (iii) to consider the insect as being “established” in the case of a high population density: if $p_{j}^{t}≃1$, then $O_{j}^{t}=2$ with probability ${\left(p_{j}^{t}\right)}^{2}≃1$.

#### Computation of a likelihood

We define the set of all the observations:

$$\text{Obs}:=\{O_{j}^{t},\;t=2004,\ldots ,2015,\;j\in J^{t}\},$$

where *J*^*t*^ is the set of departments monitored during year *t* (some departments were not monitored in 2004 and 2005, see Fig 3). The likelihood function L(Θ) is then given by:

L(Θ)=P(Obs|Θ). (14)

Using the probabilistic model (12) and the independence assumption on the observations, conditionally on the population densities $V_{j}^{t}$, the likelihood can be computed explicitly:

$$L\left(\Theta \right)=\prod_{t=2004}^{2015}\prod_{j\in J^{t}}\sum_{m=0}^{2}1\left(O_{j}^{t}=m\right)\frac{2}{m!(2-m)!}{\left(p_{j}^{t}\right)}^{m}{\left(1-p_{j}^{t}\right)}^{2-m},$$ (15)

where 1(x=y)=1 if *x* = *y* and 0 otherwise. We recall that $p_{j}^{t}$ is defined by Eq (13) and roughly corresponds to the average population density in the *j*^th^ department at time *t*.

The maximum-likelihood estimator (MLE) is the value of Θ such that the likelihood function reaches a global maximum, i.e., the value that is most likely to have produced the observations. The MLE was computed for the 2D/1D model, leading to a parameter value Θ*, and the 2D model, leading to a parameter value $\tilde{\Theta }$. To estimate these values, we used a standard quasi-Newton method minimisation algorithm applied to -ln(L(Θ)); see S3 Text for more details.

#### Model comparison and assessment of the predictive power

To compare the 2D/1D model with the classical 2D model, we computed the Bayesian information criterion (BIC) [34]. This criterion is based on the comparison of the log-likelihoods obtained with the fitted models, penalized by the number of parameters in the model. More precisely, the BIC is computed as follows:

BIC=-2ln(L)+nln(N), (16)

where L is the value of the likelihood function corresponding to the MLE Θ* (resp. $\tilde{\Theta }$ for the 2D model), *n* is the number of parameters (*n* = 6 for the 2D/1D model and *n* = 3 for the 2D null model), and *N* = 959 is the number of observations used in the computation of the likelihood (964 minus the 5 observations of 2003, which were already involved in the construction of the initial condition). The lower BIC score indicates a better model.

To assess the goodness of fit and predictive power of the two models, and because the observation data are of discrete type, the multi-category Brier score [35–37] was also calculated. For data including three categories (here, 0 = absent, 1 = introduced and 2 = established), the half-Brier score is defined as follows, for each year *t* in the period 2004–2015:

$$BS\left(t\right)=\frac{1}{2\;N_{t}}\sum_{j\in J^{t}}\sum_{m=0}^{2}{\left(P_{j}^{t}\left(m\right)-1\left(O_{j}^{t}=m\right)\right)}^{2},$$ (17)

where *N*_*t*_ is the number of observations (number of observed departments) during year *t* and $P_{j}^{t}\left(m\right)$ is the “forecast probability”, i.e., the probability that event *m* occurs at time *t* and in department *j* using the present model. In our case,

$$P_{j}^{t}\left(m\right)=\frac{2}{m!(2-m)!}{\left(p_{j}^{t}\right)}^{m}{\left(1-p_{j}^{t}\right)}^{2-m}.$$

The half-Brier score *BS* is a mean squared error measure of probability forecasts over sample Obs. The score *BS* has a range of 0 to 1; *BS* = 0 corresponds to a perfect forecast (all the true events were forecast with probability 1), and *BS* = 1 corresponds to the worst score (for each couple (*t*, *j*), probabilities 1 were associated with events which did not happen).

To further assess model performance, we computed the Brier skill score (*BSS*) [38] for the 2D/1D model. The *BSS* is defined as

$$BSS\left(t\right)=1-\frac{BS(t)}{BS_{\text{ref}}\left(t\right)},$$ (18)

where *BS*_ref_(*t*) is the Brier score of the 2D null model. The *BSS* reflects the relative gain in the predictive power of a model, compared to a null model (here the 2D model) where the roads are not taken into account. *BSS* = 1 corresponds to a perfect forecast, *BSS* = 0.5 to a twice better performance than the reference model and *BSS* = 0 to the same performance as the reference model. Negative values would indicate that the 2D/1D model is less accurate than the reference 2D model.

### Solving the 2D and 2D/1D models: numerical and algorithmic aspects

The simulations were carried out using a finite-element method in space and an implicit scheme in time. The non-linearity was dealt with using a Newton-Raphson algorithm. The simulations were performed using the Freefem++ finite-element framework [39]. The average computation time for one simulation, over the whole period (2003, 2015) was of 10 minutes for the 2D/1D model and 5 minutes for the 2D model, on an Intel(R) Core(TM) i7-2720QM CPU @ 2.20GHz. For more details on the numerical and algorithmic aspects, see S1 and S2 Figs. The source code of the solver is available in S1 File.

For both the 2D and 2D/1D models, the following constraints on the parameter values were assumed:

- Growth rate *r*. Consider a Malthusian non-spatial model *v*′ = *rv*, corresponding to the absence of intra-specific interactions and of dispersion. During one year, which corresponds to one unit of time in these computations, the population increases by a factor *e*^*r*^. Assuming that this factor is in the range (2, 100), we assume that *r* ∈ (ln(2), ln(100)).
- 2D diffusion coefficient *d*. In the numerical computations, one unit of length corresponds to 110 km. An average speed between 0.1 m per minute and 30 m per minute is assumed. Assuming a random walk movement with one direction change per minute, and using the formula [15, 24]:
  $$d=\frac{{\left(\text{length}\;\text{of}\;\text{a}\;\text{straight-line}\;\text{move}\;\text{during}\;\text{one}\;\text{time}\;\text{step}\right)}^{2}}{4\times \text{duration}\;\text{of}\;\text{the}\;\text{time}\;\text{step}},$$
  approximate bounds are obtained for *d*: *d* ∈ (10^−7^, 10^−2^).
- 1D diffusion coefficient *D*. Assuming a random walk movement with one direction change per hour and using the 1D formula
  $$D=\frac{{\left(\text{length}\;\text{of}\;\text{a}\;\text{straight-line}\;\text{move}\;\text{during}\;\text{one}\;\text{time}\;\text{step}\right)}^{2}}{2\times \text{duration}\;\text{of}\;\text{the}\;\text{time}\;\text{step}},$$
  we assume that *D* ∈ (10^−1^, 10^4^), corresponding approximately to an average speed between 0.5 km/hour and 105 km/hour.
- 1*D* → 2*D* exchange rate *ρ*_12_. The quantity 1/*ρ*_12_ can be interpreted as the expected time spent in the 1*D* part of the domain before going back to the 2D part. It is assumed that *ρ*_12_ ∈ (2 ⋅ 10^3^, 6 ⋅ 10^3^), corresponding to an expected time between 1.5 hour and 4.4 hours.
- 2*D* → 1*D* exchange rate *ρ*_21_. Let *V*(*t*) be the number of individuals situated in a *l*_1_ m wide and *l*_2_ m long strip along a 1D element. In the absence of transfers from and into this strip other than with the 1D element, *V*(*t*) approximately satisfies *V*(*t* + *δ*_*t*_)/*V*(*t*) = exp(−*ρ*_21_ *δ*_*t*_/*l*_2_). Assuming that between 0.1% and 50% of the individuals situated in a 20 m wide strip are transferred to the 1D element in *δ*_*t*_ = one day, the result is *ρ*_21_ ∈ (7 ⋅ 10^−5^, 5 ⋅ 10^−2^).
- Source terms *u*_s_, *v*_s_. It is assumed that *u*_s_, *v*_s_ ∈ (10^−6^, 1), with the lower bound of this interval corresponding to almost no individual and the upper bound to the carrying capacity.

## Results

### Simulation results

The MLE Θ* for the 2D/1D model is

$$\left\{r^{*},d^{*},D^{*},\rho_{12}^{*},\rho_{21}^{*},u_{\text{s}}^{*}\right\}=\left\{1.3,8.3\cdot 10^{-4},9.8\cdot 10^{2},4.3\cdot 10^{3},3.3\cdot 10^{-2},5.8\cdot 10^{-3}\right\}.$$

**Fig 5:**
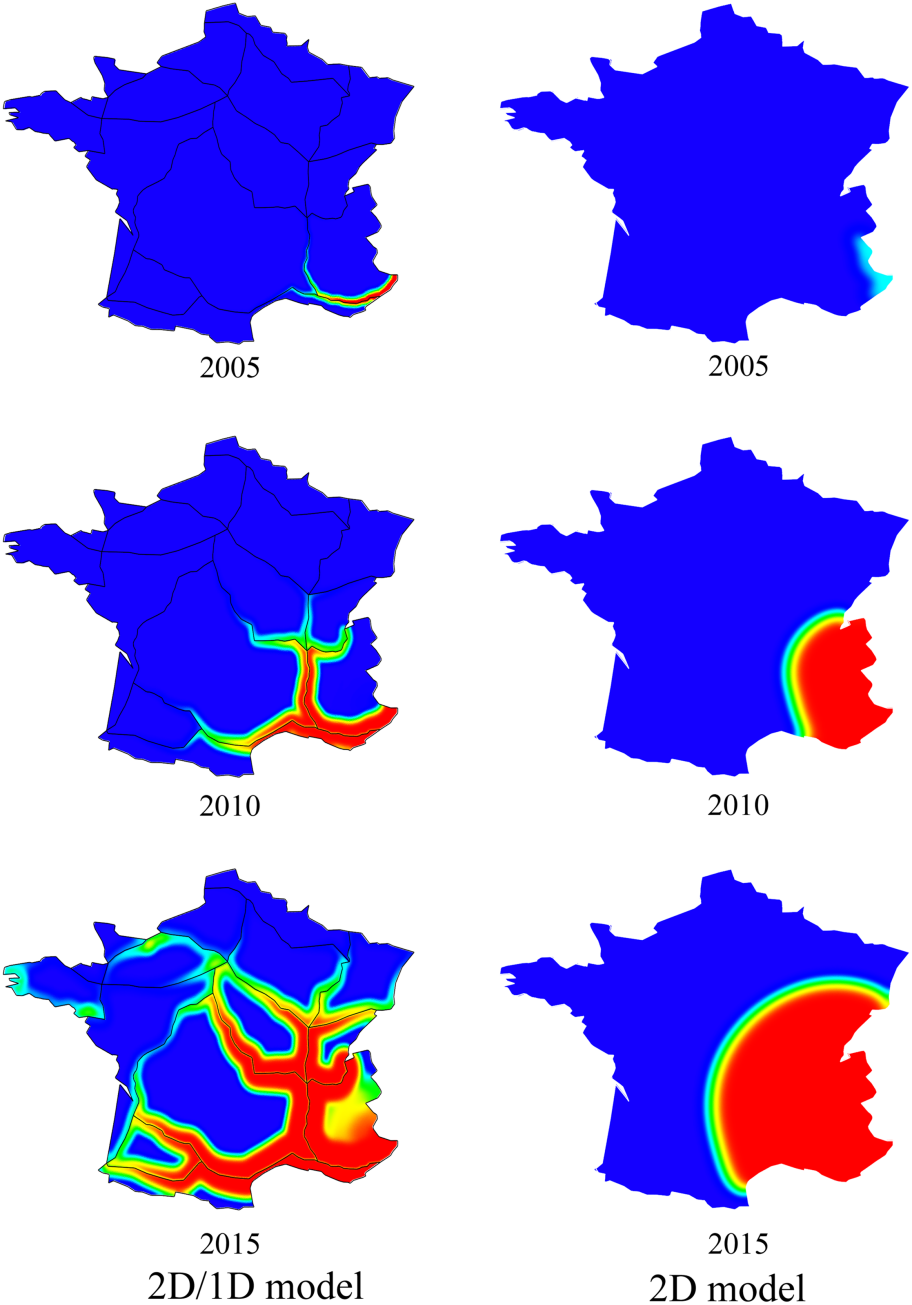
Simulation results. Left: dynamics of the mosquito population density corresponding to the solution of the 2D/1D model with the parameter value Θ* given by maximum-likelihood estimation. Right: dynamics of the mosquito population density given by the 2D model with the maximum-likelihood estimator $\tilde{\Theta }$. Values range from 0 (blue) to 1 (carrying capacity, red).

The population densities corresponding to the solution of the 2D/1D model with Θ* are depicted in Fig 5 (left) at several observation times; see also the video file in S1 Video. With these parameter values, the population density is far from homogeneous, with faster propagation along highways, followed by diffusion into surrounding departments.

The MLE $\tilde{\Theta }$ for the 2D model is $\left\{\tilde{r},\tilde{d},{\tilde{v}}_{\text{s}}\right\}=\left\{4.4,8.3\cdot 10^{-3},10^{-6}\right\}$. The corresponding population densities are depicted in Fig 5 (right); a video file is also available (S2 Video). The obtained pattern is clearly different than in the 2D/1D case; with the 2D model, the wave of colonisation propagates concentrically from the introduction region.

Using the same arguments as those developed in the previous section, we can get a mechanistic interpretation of some parameter values. The parameter values corresponding to MLE Θ* (resp. $\tilde{\Theta }$ for the 2D model) can be interpreted as follows:

- r* = 1.3 (resp. $\tilde{r}=4.4$): the population would increase by a factor 3.7 (resp. 81) per year in the absence of competition;
- *d* = 8.3 ⋅ 10^−4^ (resp. $\tilde{d}=8.3\cdot 10^{-3}$): with one direction change per minute, the length of a one-minute straight-line move in the matrix is about 9m (resp. 27m);
- *D* = 9.8 ⋅ 10^2^: with one direction change per hour, the length of a one-hour straight-line move on a highway is about 52 km.

Although the speed in the matrix and the growth rate corresponding to the MLE are larger in the 2D case than in the 2D/1D model, the northward expansion is clearly faster with the 2D/1D model. This shows that the presence of the corridors with faster diffusion clearly enhances the rate of range expansion, as already observed in the theoretical model of Berestycki et al. [16].

### Model fit and predictive power

#### Model fit

We check here that the 2D/1D model with the MLE Θ* gives a good fit to the data. In that respect, we first compare the maximum log-likelihood values obtained with the 2D/1D model and the classical 2D model: $-\ln(L\left(\Theta^{*}\right))=365$ for the 2D/1D model and $-\ln(L\left(\tilde{\Theta }\right))=3037$ for the 2D null model. To confirm this strong evidence for the 2D/1D model, we compute the Bayesian information criterion (BIC, see formula (16)) for the two models. Again, the 2D/1D model obtains a lower BIC (771 vs 6094), indicating a better fit than the 2D model.

#### Predictive power

To assess the predictive power of the 2D/1D model, we split the data set Obs into two parts. One part, corresponding to the period 2004–2012 was used as a training series, to compute new MLEs $\Theta_{\text{train}}^{*}$ and ${\tilde{\Theta }}_{\text{train}}$ for the 2D/1D and 2D models, respectively. The other part of the data set, corresponding to the period 2013–2015, was used as a “test set” to check the performances of the two models.

The relative performances of the 2D/1D model compared to the 2D model during the test period 2013–2015 are assessed through the Brier skill score (*BSS*, see formula (18)): *BSS*(2013) = 0.46, *BSS*(2014) = 0.44, and *BSS*(2015) = 0.49, indicating a far better predictive power of the 2D/1D model.

**Fig 6:**
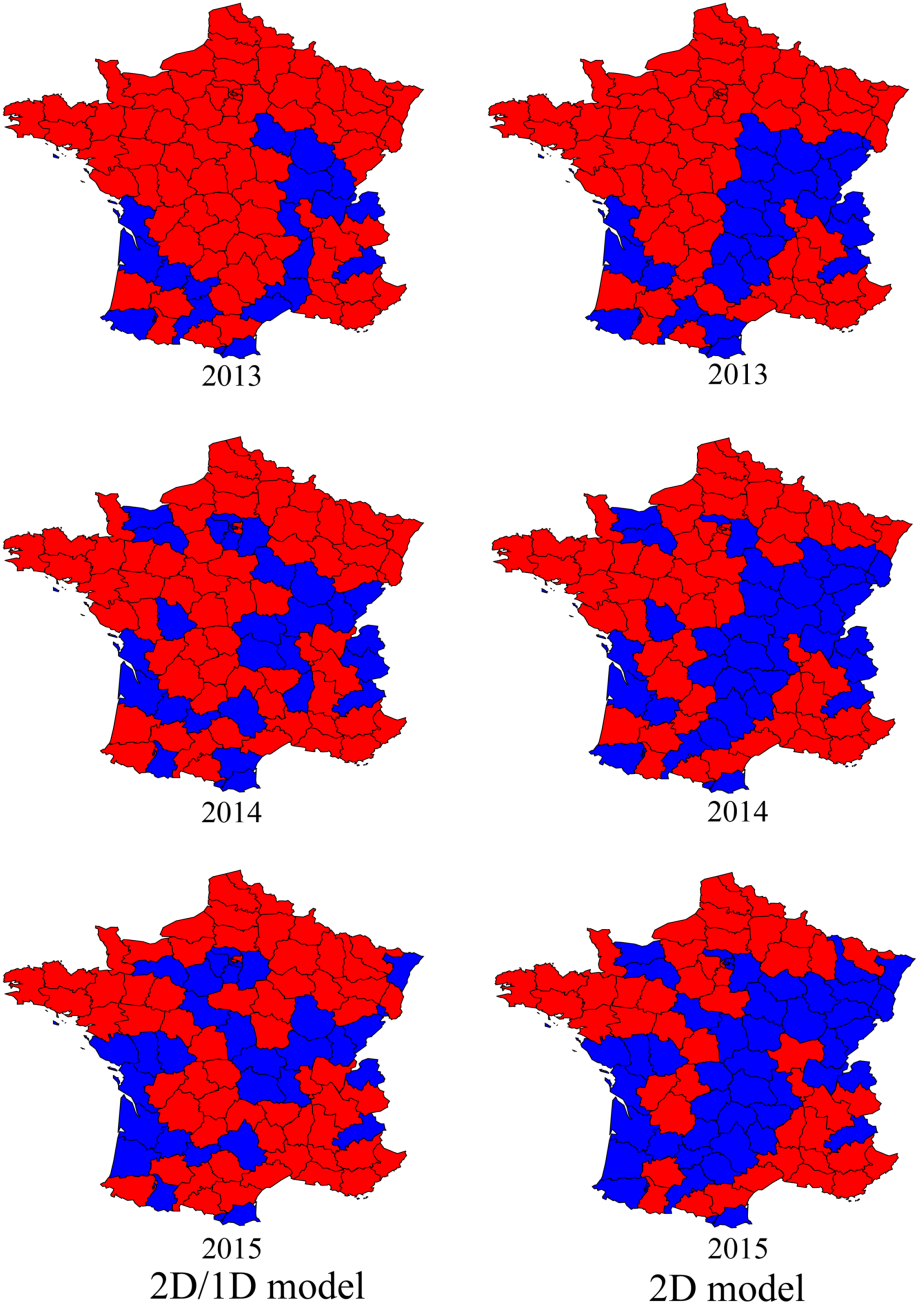
Predictive power. Red departments indicate a good prediction: the event (0 = absent, 1 = introduced or 2 = established) which was predicted with the highest probability is the event which actually occurred. Blue departments indicate an incorrect prediction: the event which was predicted with the highest probability is not the event which actually occurred. The model parameters $\Theta_{\text{train}}^{*}$ (2D/1D model) and ${\tilde{\Theta }}_{\text{train}}$ (2D model) were computed by maximum-likelihood estimation, based on the data of the training period 2004–2012.

To visualise the local predictive performance of the 2D/1D model, we depicted in each department during the test period 2013–2015 whether the event (0 = absent, 1 = introduced or 2 = established) which was predicted with the highest probability is the event that actually occurred (Fig 6). With the 2D/1D model, the rate of correct prediction (number of departments where the true event was predicted/total number of departments) is relatively stable: 81% in 2013, 69% in 2014 and 66% in 2015, indicating that acceptable predictions might be obtained over a longer time horizon. On the other hand, it decreases rapidly for the 2D model: 72% in 2013, 62% in 2014 and 50% in 2015.

## Discussion

In this paper, we propose a general approach based on a system of coupled 2D/1D reaction-diffusion equations for modelling species dynamics in a landscape made up of a 2D homogeneous matrix crossed by 1D corridors. Using the example of the range expansion of *A. albopictus* in France with its main highways as 1D elements, we have shown that this approach can be applied to realistic landscapes and data. Because the implementation of the domain only requires the geometrical characteristics of the polygonal patches as inputs, this approach can be adapted to a wide range of landscapes.

To fit the model to the observed data of the range expansion of *A. albopictus*, we have coupled the 2D/1D model with a probabilistic observation model. The resulting mechanistic-statistical model was fitted to data by maximum-likelihood estimation (Bayesian methods could have been used as well; see, e.g. [20–22]). The 2D/1D approach demonstrated a better fit and a higher predictive power (assessed by cross-validation) than a classical 2D reaction-diffusion approach, thus emphasizing the importance of taking into account the road network in the expansion of *A. albopictus*. Regarding the dynamics of that species, this conclusion is consistent with recent findings [5].

One of the interests of such mechanistic approaches, compared to more empirical statistical approaches, is that they lead to biologically meaningful parameter values. Here, we have been able to estimate the growth rate of *A. albopictus* and its dispersal rate in the matrix and in the corridors. The estimates were clearly different from those obtained with a classical 2D diffusion model. The better fit obtained with the 2D/1D model suggests that one can be more confident with the parameter values obtained using this approach. These parameters may then be used in other studies, e.g., based on more realistic and more complex stochastic individual-based approaches, were parameter values cannot be easily estimated.

In the application considered here, we assumed that the roads did not act as barriers for *A. albopictus* (*α* >> 1). In other applications, barrier effects are known to play an important role, especially in species conservation studies [8]. A possible application of our approach is the estimation of the permeability parameter *α*, with possible spatial heterogeneities in its values depending on the nature of the corridors. This could lead to a classification of the strength of the barrier effect, depending on the nature of the barrier (road, hedgerow, river,…) and on the considered species.

Traditionally, space-dependent mobility is taken into account through heterogeneous diffusion terms in scalar reaction-diffusion equations, e.g., ∂_*t*_ *v* = Δ(*d*(*x*, *y*)*v*) + *f*(*v*), with different values of *d*(*x*, *y*) depending on whether (*x*, *y*) is part of a highway or not (see, e.g., [12–15] for examples of such scalar reaction-diffusion equations in heterogeneous media). However, this approach would have several numerical drawbacks compared to the 2D/1D system proposed here. Highways are about 25m wide, which corresponds to 1/40000^th^ of the domain’s width in the present case. This would lead either to an extremely fine mesh in the finite-element method and therefore to very long computational times, or to an unbalanced mesh, leading to an ill-conditioned linear system [40]. In the proposed approach, the thicknesses of the highways are not taken into account; hence, they have no effect on the mesh size.

Nevertheless, reaction-diffusion equations with heterogeneous coefficients are certainly more appropriate when the heterogeneities do not correspond to linear elements in the landscape. A natural extension of this work would thus be to include spatially heterogeneous dispersal or reproduction terms in the 2D and/or the 1D part of the model. This extension would allow, in more empirically-oriented studies, taking into account factors which are known to play an important role in species dynamics, such as land use or climate conditions in the case of *A. albopictus*[5, 41]. Taking climate conditions into account will certainly be of critical importance to maintain a good predictive power as the species reach higher latitudes, where climate constraints will be stronger.

The proposed framework could also be extended to other types of models, for instance 1D models including transport terms to account for directional flows and give a better description of biased movements, e.g., in hydrological networks. Time-dependent parameters could be added as well, e.g., to describe seasonal variations in vehicle flows.

Corridors of movement are known to play a key role on predator-prey interactions. Mckenzie et al. [42] studied the effect of corridor density (seismic lines in their case) on the encounter rate of wolves with their prey. A modelling approach based on elliptic partial differential equations (PDE) describing the average time required for a predator to locate a stationary prey enabled them to show that the presence of corridors can lead to an increase in prey encounters. Our 2D/1D model could be extended to handle several interacting species, by replacing the scalar 2D and 1D equations by systems of reaction-diffusion equations. The extension of our approach to predator-prey models with diffusion terms [13, 43] might find applications in biological control, where one typically looks for agricultural landscape structures which enhance pest (= prey) regulation by natural enemies (= predators) [44]. This may help to understand the effect of linear elements such as field margins and hedgerows which often constitute refuge habitats for natural enemies of crop pests. Our approach could also be applied to epidemic models such as SIR models [25]. This would be especially relevant for epidemics such as cholera whose transmission is mediated by water: the disease can spread through waterways and river networks. A network-based approach and its 1D PDE approximation has already been proposed in [45]; a possible extension would be to adapt their model and embed the network into a 2D matrix containing the susceptible populations.

## Supporting Information

S1 Text Description of the coordinate transformation. (PDF)

S2 Text Proof of the conservation of the total population. (PDF)

S3 TextA quasi-Newton algorithm for minimising the log-likelihood function -ln(L(Θ)).(PDF)

S1 Fig Triangular mesh used for solving the 2D/1D model. The simulation of the 2D/1D models (2), (3) and (4), with the boundary conditions Eqs (6)–(9), and with the above-specified domain (Fig 4, left) and forms of the functionals Eq (10) and *g* = 0 was computed for any given Θ = {*r*, *d*, *D*, *ρ*_12_, *ρ*_21_, *u*_s_} using a mesh composed of 65742 nodes. A sparse block matrix was built for each Newton-Raphson iteration. The diagonal blocks contained the reaction-diffusion formulation for each 2D and 1D domain. The extra-diagonal blocks corresponded to the interactions between the domains. This led to a problem with 70570 unknowns. The average computation time for one simulation was of 10 minutes.
(PDF)

S2 Fig Triangular mesh used for solving the 2D model. The simulation of the classical 2D reaction-diffusion model (11) for any given Θ = {*r*, *d*, *v*_s_} was based on a mesh composed of 71325 nodes on the domain presented in Fig 4. The average computation time for one simulation, over the whole period (2003, 2015) on was of 5 minutes.
(PDF)

S1 File Source code of the 2D/1D solver. The Freefem++ programs build sparse matrices corresponding to the variational formulations of the 1D and 2D reaction-diffusion equations and to the interactions between all of the 2D and 1D domains. The second step consists in assembling these elementary matrices into a sparse block matrix which is used in the Newton-Raphson algorithm. The programs (ZIP) are listed below: *simul.edp* is the main program; *buildMatGen.edp* builds the sparce block matrix; *defDofsGen.edp* defines the degree of freedom of each domains; *defGeomGen.edp* builds the meshes from the geometry; *defICGen.edp* contains the initial conditions; *defVarfGen.edp* contains the variational formulations; *defMatGen.edp* gets index of degree of freedom shared between consecutive edges; *defParamsGen.edp* declares the parameters of the model; *inputParam.edp* contains the parameter values; *myMacro.edp* contains a macro to compute the likelihood; *postTraitement.edp* computes the likelihood of the current state; *postTraitementEnd.edp* computes the likelihood of the simulation; *postTraitementInit.edp* initializes the likelihood; *postTraitementLoad.edp* loads the meshes used for the likelihood computation; *timeSteppingGen.edp* does the implicit time step; *mat1D2Duseful.edp* contains some macro to build the elementary blocks from the variational formulation; *geomMacro.edp* contains a macro getting the boundary degrees of freedom located on a edge; *resizeCSR.cpp* is a C++ program which modifies the sparse block matrix to implement the boundary conditions between 1D edges.
(ZIP)

S1 Video Simulation of the spatio-temporal dynamics of the density of *A. albopictus* from 2003 to 2015 with the 2D/1D model. The parameters correspond to the MLE.
(AVI)

S2 Video Simulation of the spatio-temporal dynamics of the density of *A. albopictus* from 2003 to 2015 with the 2D model. The parameters correspond to the MLE.
(AVI)
