# Supplementary material for: Modelling Population Dynamics in Realistic Landscapes with Linear Elements: A Mechanistic-Statistical Reaction-Diffusion Approach
Source: PLoS One. 2016 Mar 17;11(3):e0151217. doi: 10.1371/journal.pone.0151217 (PMC4795701; doi:10.1371/journal.pone.0151217)
Supplement: S1 Text — (PDF) [file pone.0151217.s001.pdf]

Polygonal shapes. To state a simple equation for the 1D dynamics on the edges, a change of variable was defined, which transformed each edge λ into an interval $(0, L(\lambda))$, where $L(\lambda)$ was the length of the edge λ. That is, if λ connects two vertices $\underline{\mathbf{s}}(\lambda) \in \mathbb{R}^2$ and $\overline{\mathbf{s}}(\lambda) \in \mathbb{R}^2$, one can write:

$$\lambda := \{\underline{\mathbf{s}}(\lambda) + z\,(\overline{\mathbf{s}}(\lambda) - \underline{\mathbf{s}}(\lambda))/L(\lambda),\ z \in (0, L(\lambda))\}. \tag{A}$$

Hence, for each $(x, y) \in \lambda$, up to the change of variable

$$(x, y) = \underline{\mathbf{s}}(\lambda) + z\,(\overline{\mathbf{s}}(\lambda) - \underline{\mathbf{s}}(\lambda))/L(\lambda), \tag{B}$$

it is possible to write

$$u(t, x, y) = \tilde{u}(t, z),\ z \in (0, L(\lambda)).$$

Non-polygonal shapes. For the sake of clarity, the proposed approach was presented in the main text for a polygonal domain Ω and polygonal patches Ω_i. This approach can be extended to more general geometries by replacing the edges λ_i^k by arcs which are not necessarily straight-line segments. The only change in the framework described above is the change of variable (B), which must be generalised. Consider an arc λ of length $L(\lambda)$, parameterised by a given function $j(z):\ [0, L(\lambda)] \to \mathbb{R}^2$ satisfying

$$\int_0^{L(\lambda)} \|dj/dz\|\, dz = L(\lambda),$$

where $\|\cdot\|$ is the Euclidian norm. The generalisation of (B) is simply $(x, y) = j(z)$.
