# Supplementary material for: Modelling Population Dynamics in Realistic Landscapes with Linear Elements: A Mechanistic-Statistical Reaction-Diffusion Approach
Source: PLoS One. 2016 Mar 17;11(3):e0151217. doi: 10.1371/journal.pone.0151217 (PMC4795701; doi:10.1371/journal.pone.0151217)
Supplement: S2 Text — (PDF) [file pone.0151217.s002.pdf]

We assume that the growth functions satisfy $f = 0$ and $g = 0$, and that there is no introduction point (we recall that reflecting conditions have been assumed on the exterior boundaries). Let us define

$$P(t) = P_{2D}(t) + P_{1D}(t),$$

where $P_{2D}$ is the total population in the 2D patches:

$$P_{2D}(t) = \sum_{i=1}^{I} \int_{\Omega_i} v_i(t, \cdot), \tag{A}$$

and $P_{1D}$ is the total population on the 1D edges:

$$P_{1D}(t) = \sum_{\lambda_i^k \in \Lambda_{\text{int}}} \int_{\lambda_i^k} u_i^k(t, \cdot), \tag{B}$$

where $\Lambda_{\text{int}}$ corresponds to the set of interior edges (corridors).

Integrating by parts the equation (2), which is satisfied by the population density on any patch $\Omega_i$, the following can be obtained:

$$\partial_t \int_{\Omega_i} v_i = \int_{\Omega_i} \Delta v_i = \int_{\partial\Omega_i} \nabla v_i \cdot \mathbf{n}. \tag{C}$$

Using the boundary conditions (3) and (4), and because

$$\partial\Omega_i = \bigcup_k \lambda_i^k,$$

the result is:

$$\partial_t \int_{\Omega_i} v_i = d \sum_{k \text{ s.t. } \lambda_i^k \in \Lambda_{\text{int}}} \mu \int_{\lambda_i^k} u_i^k - \nu \int_{\lambda_i^k} v_i. \tag{D}$$

Using the relations (A) and (D), it can be observed that:

$$P'_{2D}(t) = \sum_{\lambda_i^k \in \Lambda_{\text{int}}} \mu \int_{\lambda_i^k} u_i^k - \nu \int_{\lambda_i^k} v_i. \tag{E}$$

Then, rewriting $P_{1D}$ in the form

$$P_{1D}(t) = \sum_{\lambda_i^k \in \Lambda_{\text{int}}} \int_0^{L(\lambda_i^k)} \tilde{u}_i^k(t, \cdot)$$

and using the equation (5), we get:

$$\begin{aligned} P'_{1D}(t) = \sum_{\lambda_i^k \in \Lambda_{\text{int}}} \quad & D(\partial_z \tilde{u}_i^k(L(\lambda_i^k)) - \partial_z \tilde{u}_i^k(0)) \\ & -\mu \int_{\lambda_i^k} u_i^k + \nu \int_{\lambda_i^k} v_i \\ + \sum_{\lambda_i^k \in \Lambda_{\text{int}}} \quad & -\alpha \int_{\lambda_i^k} u_i^k + \alpha \int_{\lambda_i^k} u_{i'}^{k'}. \end{aligned} \tag{F}$$

Using the standard boundary conditions (6) and (8), for each $i$,

$$\sum_{k \text{ s.t. } \lambda_i^k \in \Lambda_{\text{int}}} D(\partial_z \tilde{u}_i^k(L(\lambda_i^k)) - \partial_z \tilde{u}_i^k(0)) = 0.$$

In addition, because the application $Q: \ (i,k) \mapsto (i',k')$ is a bijection from the set of all indices $\{(i,k) \ \text{ s.t. } \lambda_i^k \in \Lambda_{\text{int}}\}$ to itself, it can readily be determined that

$$\sum_{\lambda_i^k \in \Lambda_{\text{int}}} \alpha \int_{\lambda_i^k} u_i^k = \sum_{\lambda_i^k \in \Lambda_{\text{int}}} \alpha \int_{\lambda_i^k} u_{i'}^{k'}.$$

Finally, adding the equations (E) and (F) results in:

$$P'(t) = 0,$$

which means that the total mass was conserved.
