# Supplementary material for: Modelling Population Dynamics in Realistic Landscapes with Linear Elements: A Mechanistic-Statistical Reaction-Diffusion Approach
Source: PLoS One. 2016 Mar 17;11(3):e0151217. doi: 10.1371/journal.pone.0151217 (PMC4795701; doi:10.1371/journal.pone.0151217)
Supplement: S3 Text — (PDF) [file pone.0151217.s003.pdf]

The computation of the MLEs was based on numerical minimisation of the log-likelihood function $-\ln(\mathcal{L}(\Theta))$. We used a deterministic algorithm, the BFGS (Broyden-Fletcher-Goldfarb-Shanno) quasi-Newton method with a cubic line search procedure, which was implemented using the MATLAB® fmincon function. We repeated the minimization algorithm 30 times, with 30 randomly chosen initial guesses. For each minimization, the BFGS needed around 400 simulations on average.
