# Supplementary figures and images for: Modelling Population Dynamics in Realistic Landscapes with Linear Elements: A Mechanistic-Statistical Reaction-Diffusion Approach
Source: PLoS One. 2016 Mar 17;11(3):e0151217. doi: 10.1371/journal.pone.0151217 (PMC4795701; doi:10.1371/journal.pone.0151217)

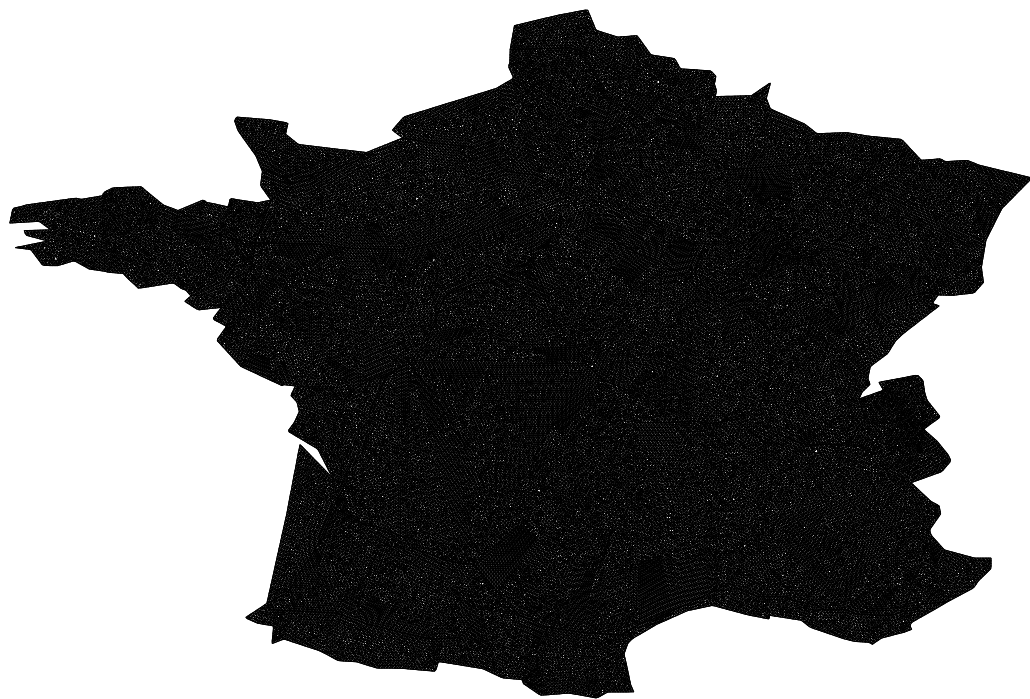

Supplement: S1 Fig — The simulation of the 2D/1D models (2), (3) and (4), with the boundary conditions Eqs (6)–(9), and with the above-specified domain (Fig 4, left) and forms of the functionals Eq (10) and g = 0 was computed for any given Θ = {r, d, D, ρ12, ρ21, us} using a mesh composed of 65742 nodes. A sparse block matrix was built for each Newton-Raphson iteration. The diagonal blocks contained the reaction-diffusion formulation for each 2D and 1D domain. The extra-diagonal blocks corresponded to the interactions between the domains. This led to a problem with 70570 unknowns. The average computation time for one simulation was of 10 minutes. (PDF) [file pone.0151217.s004.pdf]

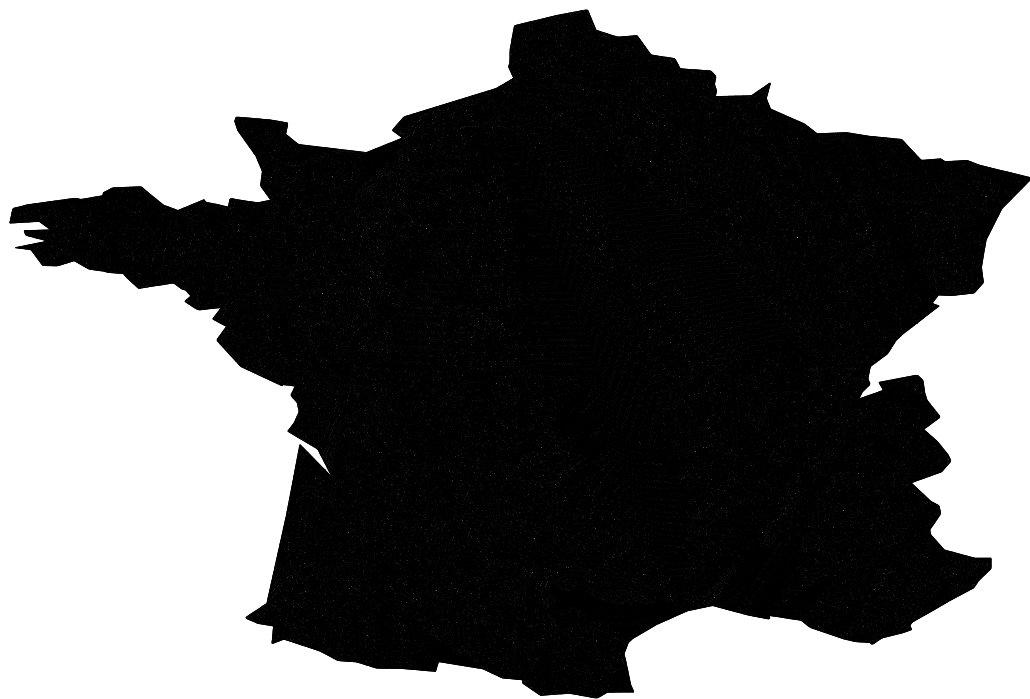

Supplement: S2 Fig — The simulation of the classical 2D reaction-diffusion model (11) for any given Θ = {r, d, vs} was based on a mesh composed of 71325 nodes on the domain presented in Fig 4. The average computation time for one simulation, over the whole period (2003, 2015) on was of 5 minutes. (PDF) [file pone.0151217.s005.pdf]
